# Liver and brain abscess caused by *Aggregatibacter paraphrophilus *in association with a large patent foramen ovale: a case report

**DOI:** 10.1186/1752-1947-4-69

**Published:** 2010-02-24

**Authors:** Shaumya Ariyaratnam, Parag R Gajendragadkar, Richard J Dickinson, Phil Roberts, Kathryn Harris, Andrew Carmichael, Johannis A Karas

**Affiliations:** 1 Department of Medicine, Hinchingbrooke Health Care NHS Trust, Hinchingbrooke Hospital, Hinchingbrooke Park, Huntingdon, PE29 6NT, UK; 2 Department of Microbiology, Level 4 Camelia Botnar Laboratories, Great Ormond Street Hospital for Children NHS Trust, Great Ormond Street, London, WC1N 3JH, UK; 3 Department of Infectious Diseases, Addenbrooke's Hospital, Cambridge University Hospitals NHS Foundation Trust, Hills Road, Cambridge, CB2 0QQ, UK; 4 Health Protection Agency, East of England, Microbiology Laboratory, Papworth Hospital, Ermine Road, Papworth Everard, CB23 3RE, UK

## Abstract

**Introduction:**

*Aggregatibacter paraphrophilus *(former name *Haemophilus paraphrophilus*) is a normal commensal of the oral flora. It is a rare cause of hepatobiliary or intracerebral abscesses.

**Case presentation:**

We report a case of a 53-year-old Caucasian man with a liver abscess and subsequent brain abscesses caused by *Aggregatibacter paraphrophilus*. The probable source of the infection was the oral flora of our patient following ingestion of a dental filling. The presence of a large patent foramen ovale was a predisposing factor for multifocal abscesses.

**Conclusion:**

In this case report, we describe an unusual case of a patient with both liver and brain abscesses caused by an oral commensal *Aggregatibacter paraphrophilus *that can occasionally show significant pathogenic potential.

## Introduction

*Aggregatibacter paraphrophilus *(former name *Haemophilus paraphrophilus*) is a species of Gram-negative coccobacilli formerly in the genus Haemophilus, now Aggregatibacter [1]. It is a normal commensal of the human oral cavity and pharynx. It is documented as being a rare cause of subacute bacterial endocarditis, brain abscess, sinusitis, arthritis and osteomyelitis and is often associated with recent dental treatment [2]. Diagnosis unfortunately is hindered by its fastidious and slow-growing nature [3].

Here we describe a rare case of a patient with both liver and brain abscesses caused by *Aggregatibacter paraphrophilus*, incidentally found to have a patent foramen ovale.

## Case presentation

A 53-year-old Caucasian man presented with a five-day history of malaise, productive cough, fever and rigors. He had been treated by his primary care doctor for two days with oral clarithromycin without improvement. He had undergone dental root canal surgery two months previously; the dental filling fell out the day before admission and our patient may have accidentally swallowed it. He never injected drugs intravenously or received blood transfusion. He never smoked, rarely drank alcohol and took no other medication. On examination, he had a fever of 39°C, blood pressure of 132/68 mmHg, sinus tachycardia of 110 beats per minute. Auscultation of the chest revealed some crackles at the right lung base. His heart sounds were normal, and abdominal examination was normal.

The haemoglobin level was 13.0 g/dL (mean corpuscular volume of 85fl); the platelet count was 84 × 10^9^/L; the white cell count 10.0 × 10^9^/L, with a neutrophilia of 9.0 × 10^9^/L. The serum albumin was reduced at 29 g/L, bilirubin 2 micromoles/L, alkaline phosphatase 466 U/L (normal range 25 to 140) and alanine aminotransferase 239 U/L (normal range 10 to 40). The C-reactive protein (CRP) was raised at 178 mg/L. Serum urea, creatinine, electrolytes, glucose and coagulation were within normal reference ranges. Urine analysis showed nitrites, 1+ protein, 1+ bilirubin, and trace blood. The ECG showed sinus tachycardia. Chest radiography showed a prominent right hilum. Blood cultures taken on our patient after admission showed no growth.

Community-acquired pneumonia was suspected for which our patient was treated with intravenous amoxicillin-clavulanic acid 1.2 g every 8 hours and oral clarithromycin 500 mg every 12 hours. A liver ultrasound performed because of the abnormal liver function tests revealed two well-defined areas of mixed echogenicity in the right lobe of the liver measuring 49mm and 40mm in diameter. Metastatic tumor was suspected.

**Figure 1:**
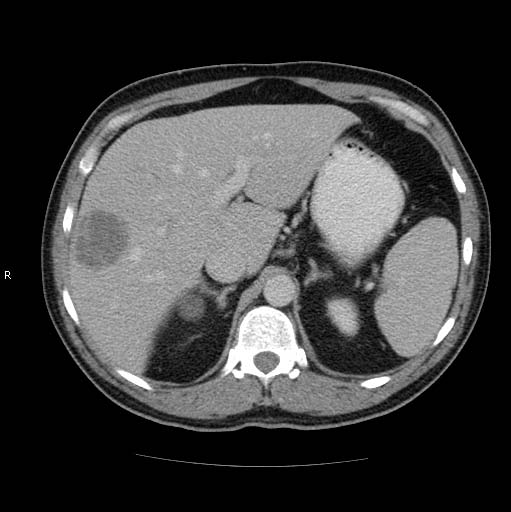
**Computed tomography of the abdomen showing a mildly enhancing peripheral hypodense lesion in liver**. Scan taken during the initial admission.

**Figure 2:**
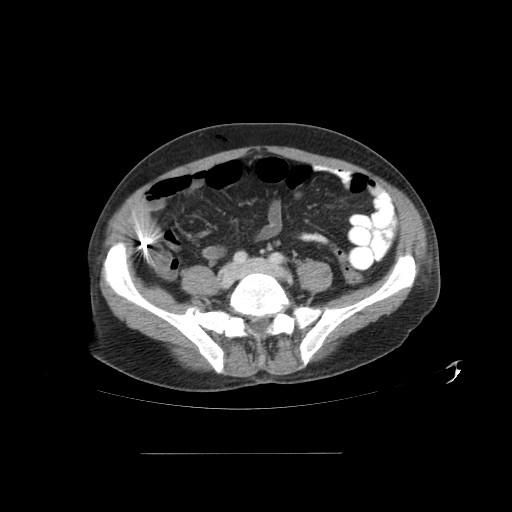
**Computed tomography of the abdomen showing a metallic artefact (likely dental amalgam) in appendix region**. Scan taken during the initial admission.

The fever of our patient continued, and on the third day, he developed a severe headache with persistent vomiting. Fundoscopy was normal. Computer tomography (CT) scanning of the head with contrast was normal. Lumbar puncture was performed which showed no white cells or red cells and no organisms identified on Gram stain or upon culture of the cerebrospinal fluid (CSF). A CT scan of the chest revealed minor basal atelectasis. A CT scan of the abdomen and pelvis revealed a single enhancing low attenuation 4.5 cm mass in the right lobe of the liver which showed some contrast enhancement [figure 1]. The other solid organs and appendix were normal, and a metal artefact was seen in the colon [figure 2].

Because he was not improving, he underwent percutaneous aspiration of the liver lesion under ultrasound guidance after six days. This drained 30 ml of pus from our patient. Gram stain showed no organisms and culture was negative. He continued to have upper abdominal pain and high fever. A repeat abdominal CT scan showed persistence of the liver abscess, and a mildly dilated appendix (approx. 12 mm diameter). Plain abdominal radiography confirmed a dense radio-opaque object consistent with amalgam dental filling in the right lower quadrant. A percutaneous pigtail drain was inserted and a further 20 ml of pus was aspirated. He was treated with intravenous ertapenem 1 g once daily and intravenous metronidazole 500 mg three times a day.

Both samples of pus that were aspirated from the liver abscess were culture negative. The causative organism was identified as *Aggregatibacter paraphrophilus *by polymerase chain reaction (PCR) amplification of the bacterial 16S ribosomal DNA followed by nucleotide sequencing, using published primers [4]. Serological tests for influenza A and B, parainfluenza, adenovirus, respiratory syncytial virus, Chlamydia, and Mycoplasma were negative. All urine, stool, cerebrospinal fluid and methicillin resistant *Staphylococcus aureus *multisite cultures were negative. A trans-thoracic echocardiogram (TTE) prior to discharge did not show evidence of endocarditis. Repeat CT scan of the abdomen after 14 days showed improvement in the liver abscess and some bilateral basal consolidation. The fever of our patient was resolved. After completing 19 days of intravenous ertapenem, it was shifted to oral amoxicillin 500 mg every eight hours for two weeks. During discharge after 29 days, his liver function tests had returned to normal, but he was anaemic with a haemoglobin of 10.7 g/dL, an erythrocyte sedimentation rate (ESR) of 94 mm/hr and CRP of 17 mg/L.

**Figure 3:**
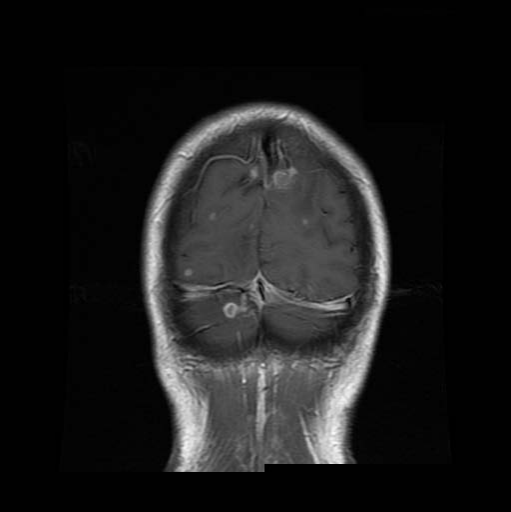
**T1 weighted magnetic resonance imaging of the head showing multiple foci of contrast enhancement (abscesses)**. Scan taken during the second admission showing lesions suspicious of abscesses near the grey-white junction of both cerebral hemispheres, and a small enhancing lesion in the right cerebellar hemisphere.

Three weeks after discharge and two weeks after having completed the course of oral amoxicillin, our patient re-presented to our hospital. Since discharge, he had been bumping into objects on his left side and for one day he had headache, rigors and a sore throat - he was re-admitted on that day 51. On examination, he was febrile with no signs of infective endocarditis. Ophthalmological examination revealed a left homonymous hemianopia with normal fundi. Repeat blood tests showed a haemoglobin of 11.4 g/dl (MCV 86.0fl) and a CRP of 62 mg/L; his renal and liver function tests were normal. A CT scan of the head with contrast performed on day 52 revealed multiple brain abscesses: a ring-enhancing lesion in the left occipital lobe and a non-enhancing low attenuation lesion in the right occipital lobe, with no mass effect. A CT scan of the abdomen showed a small resolving area of low attenuation in the liver; the appendix was normal. He was treated with intavenous meropenem 2 g every eight hours and transferred to a tertiary hospital. Magnetic resonance imaging (MRI) of the head confirmed multiple brain abscesses; there were multiple foci of contrast enhancement near the grey-white junction of both cerebral hemispheres, a more confluent area of signal change and enhancement was seen in the right occipital lobe, and a small enhancing lesion was seen in the right cerebellar hemisphere [figure 3].

On day 53, a mini-craniotomy and biopsy was performed on a left occipital ring-enhancing lesion. On microscopy, pus cells were seen but no organisms were observed on gram staining, and enriched aerobic, anaerobic and fungal cultures were negative. Results of the 16S rDNA PCR of the brain abscess biopsy again detected the sequence of *Aggregatibacter paraphrophilus*. Histopathology showed appearances typical of a brain abscess. A trans-oesophageal echo performed on day 55 showed no evidence of endocarditis but there was evidence of a patent foramen ovale (PFO) and an atrial septal aneurysm. A bubble echo was performed on day 60; during provocation by Valsalva maneuver, there was a large right-to-left shunt through the patent foramen ovale. Ultrasound scanning of the liver showed no remaining collection. Maxillo-facial assessment including dental panoramic tomography revealed no ongoing dental infection. His immunoglobulins were normal, anti-nuclear antibody and anti-neutrophil cytoplasmic antibody negative, and serological tests for human immunodeficiency virus, syphilis and toxoplasma were negative. He continued treatment with intravenous meropenem 2 g every eight hours and oral metronidazole 400 mg every eight hours added on day 54, and remained afebrile. He was discharged on day 65 since first presentation (white cell count 7.3 × 10^9^/L and CRP 5 mg/L) with intravenous ceftriaxone 2 g every 12 hours to complete four weeks of out-patient antibiotics via a peripherally inserted central line.

Follow-up CT scan of the head on day 71 showed surgical changes deep to the left occipital craniotomy; resolving right frontal and left occipital lobe abscesses; and a large hypodense area in the right occipital lobe in keeping with an established occipital infarct. Follow-up cranial MRI on day 81 revealed improvement in the size of the multiple small enhancing subcortical white matter lesions (likely microabscesses); with persistence of the right occipital infarct.

On outpatient follow-up, intravenous antibiotics were extended to complete a six week course in total; our patient was then switched to oral amoxicillin-clavulanic acid 625 mg every eight hours for a duration of two weeks. Unfortunately, his left homonymous hemianopia persisted.

Cardiology follow-up concluded that it was prudent to close the PFO as there was a possibility of further paradoxical emboli and this is planned. Our patient was put on anti-coagulant and anticonvulsant therapy and a cranial MRI on day 137 has shown further improvement of the cerebral abscesses.

## Conclusion

In this case, we highlight the potential for *Aggregatibacter paraphrophilus *to cause widespread systemic infections especially following dental treatment. Given the fastidious nature of the organism [3], it also emphasizes the value of bacterial 16S rDNA PCR amplification and sequencing in identifying bacteria in abscesses which are culture-negative as a result of prior antibiotic administration [4].

Following the root canal surgery, our patient may have developed bacterial endocarditis related to his atrial septal aneurysm and patent foramen ovale, but it was not possible to confirm this because he received treatment with antibiotics before blood cultures were taken. Minor, unrecalled trauma to the liver has been described in the literature as a predisposing factor for localisation of infection [5] and the presence of the dental filling in the colon may have given rise to the portal bacteraemia. We suspect that the shunt through the patent foramen ovale was a contributory factor in the development of the multiple brain abscesses by permitting infected material to bypass the lungs and enter the systemic circulation. The foramen ovale serves as a shunt during intrauterine life and occludes after birth with closure becoming anatomic over time. It remains patent in a small proportion of the population and is associated with embolic stroke. The association with cerebral abscess is less strong and reported only in a low number of case reports in the literature [6,7].

Another contributing factor in our patient developing brain abscesses may have been the fact that he was treated with ertapenem for his initial liver abscess. Ertapenem, unlike meropenem, is not licensed for treatment of meningitis as it exhibits wide variability in CSF/plasma ratios that preclude its use in CSF infections [8,9].

## Consent

Written informed consent was obtained from our patient for publication of this case report and any accompanying images. A copy of the written consent is available for review.

## Competing interests

The authors declare that they have no competing interests.

## Authors' contributions

SA, PG, RD, PR, AC, JK for clinical, and KH for laboratory work, all contributed to writing the article. All have read and approved the final manuscript
